# Severe bleeding complications and multiple kidney transplants in a patient with tuberous sclerosis complex caused by a novel *TSC2* missense variant

**DOI:** 10.3325/cmj.2017.58.416

**Published:** 2017-12

**Authors:** Stela Živčić-Ćosić, Karin Mayer, Gordana Đorđević, Mark Nellist, Marianne Hoogeveen-Westerveld, Damir Miletić, Sanjin Rački, Hanns-Georg Klein, Zlatko Trobonjača

**Affiliations:** 1 Department of Nephrology, Dialysis and Kidney Transplantation, Department of Internal Medicine, University Hospital Center Rijeka and Faculty of Medicine, University of Rijeka, Croatia; 2 Center for Human Genetics and Laboratory Diagnostics, Department of Molecular Genetics, Dr. Klein, Dr. Rost and colleagues, Martinsried, Germany; 3 Department of Pathology, Faculty of Medicine, University of Rijeka, Rijeka, Croatia; 4 Department of Clinical Genetics, Erasmus Medical Center, Rotterdam, the Netherlands; 5 Department of Radiology, University Hospital Center Rijeka and Faculty of Medicine, University of Rijeka, Rijeka, Croatia; 6 Department of Physiology and Immunology, Faculty of Medicine, University of Rijeka, Rijeka, Croatia

## Abstract

We presented an extremely severe case of tuberous sclerosis complex (TSC) in a female patient with recurring, life-threatening bleeding complications related to renal angiomyolipomas. Massive intratumoral hemorrhage required surgical removal of both angiomyolipomatous kidneys and kidney transplantation. During the follow-up period, the patient developed severe metrorrhagia that eventually led to hysterectomy and salpingo-oophorectomy. Bleeding from the operative sites caused the loss of the first kidney transplant received from the mother, and immediate hemorrhagic shock led to the loss of the second, cadaveric kidney allograft. The third kidney transplant had a successful outcome. Pathological analysis of all tissue specimens showed TSC-associated lesions and deformed blood vessels in the surgically removed organs. Molecular genetic analysis of *TSC1* and *TSC2* in the DNA of peripheral leukocytes identified a novel *TSC2* c.3599G>C (p.R1200P) variant. Functional assessment confirmed the likely pathogenicity of the *TSC2* c.3599G>C (p.R1200P) variant. To the best of our knowledge, this is the first report of the c.3599G>C (p.R1200P) variant in exon 29 of the *TSC2* gene related to a severe clinical course and multiple kidney transplants in a patient with TSC.

Tuberous sclerosis complex (TSC) is an inherited systemic disease caused by mutations in either the *TSC1* or *TSC2* gene (1). The *TSC1* and *TSC2* protein products, hamartin (TSC1) and tuberin (TSC2), form the TSC complex that integrates multiple signals to regulate the activity of the mechanistic target of rapamycin (mTOR) complex 1 (TORC1) and, thereby, control cell growth. Inactivation of the TSC complex, which results from the combination of a germline and somatic mutation, leads to the formation of hamartomas in multiple organs, causing highly variable clinical manifestations of the disease (2-4). TSC-associated loss of renal function is most often caused by large angiomyolipomas, which contain deformed blood vessels with a tendency to form aneurysms and bleed (5-7). Nephrectomy is usually required to stop the bleeding, followed by eventual kidney transplantation (2,3). Although end-stage renal failure is rare, kidney disease has become the leading cause of death in TSC patients (8,9). Furthermore, mortality rates in TSC patients, especially those with low cognitive function, are generally increased in comparison with that of general population (7).

We present a case of a female patient with severe TSC complicated by recurring, life-threatening postoperative bleeding episodes, whose tissue specimens from surgically removed organs were analyzed using standard pathological and immunohistological methods. The performed molecular genetic analysis of *TSC1* and *TSC2* identified a novel *TSC2* variant in the patient’s leukocyte DNA and the pathogenicity of the variant was confirmed by functional assessment (10).

## Case report

Our female patient received the diagnosis of TSC in childhood on the basis of epilepsy, learning disability, and facial angiofibromas (Table 1). In 2003, at the age of 18, the patient underwent right nephrectomy due to a giant angiomyolipoma that caused severe retroperitoneal hemorrhage. In 2006, contralateral nephrectomy with simultaneous kidney transplantation was performed due to a giant angiomyolipoma of the left kidney and hemorrhage. The patient received the kidney transplant from the mother.

**Table 1:** Timeline of the patient's clinical course

Age (year)	Clinical Course	Blood transfusions (units)		
		Erythrocyte concentrates	Fresh frozen plasma	Platelet concentrates
5 months (1985)	Epileptic seizures localized to the left arm	-	-	-
Childhood	Epilepsy, learning disability and facial angiofibromas	-	-	-
Adolescence	Angiomyolipoma of both kidneys TSC-associated brain lesions	-	-	-
18 years (2003)	Retroperitoneal hemorrhage from a giant renal angiomyolipoma followed by radical right nephrectomy		-	-
20 years (2005)	Severe retroperitoneal hemorrhage from the giant angiomyolipomatous left kidney Bronchopneumonia	4	-	-
21 years (2006)	Reccurent retroperitoneal hemorrhage followed by left nephrectomy with simultanous living kidney transplantation Uroinfections, bronchopneumonia Ovarian cysts, recurrent prolonged metrorrhagia	-	-	-
25 years (2010)	Severe metrorrhagia with worsening of allograft function followed by hysterectomy and left salpingo-oophorectomy Postoperative hemorrhage causing hydronephrosis, loss of allograft function and hemorrhagic shock followed by urgent transplantectomy Massive postoperative hemorrhage and sepsis Start of dialysis treatment	62 (17 filtered)	21	48
26 years (2011)	Second kidney transplantation and severe postoperative bleeding followed by transplantectomy Sepsis (*Haemophilus influenzae*)	38 filtered	12	-
25-28 years (2010-2013)	Frequent readmissions due to central venous catheter-related complications	2 filtered	-	-
28 years (2013)	Third kidney transplantation with severe intra- and postoperative hemodynamic instability requiring high doses of norepinephrine Bacteremia and accelerated acute cellular rejection Patient recovery and excellent graft function	5 filtered	-	-
Total		107 (54 filtered)	33	48

In 2010, the patient underwent hysterectomy with unilateral salpingo-oophorectomy due to prolonged metrorrhagia unresponsive to medical treatment and massive ovarian edema. Bleeding from the operation site caused hemorrhagic shock and kidney allograft loss. After recovery, then 26-year-old patient underwent urgent cadaveric kidney transplantation due to the imminent loss of dialysis access and non-compliance with dialysis treatment. Again, a severe bleeding episode from the operative site occurred resulting in arterial thrombosis of the allograft and transplantectomy. During the following 1.5 years, the patient was frequently readmitted due to the central venous catheter-related complications, such as bacteremia, clotting, and self-removal of the catheter due to non-compliance. Because of mental retardation, fear, and non-compliance, all catheter manipulations and diagnostic or treatment procedures had to be performed under general anesthesia. It was not possible to create and use an arteriovenous fistula as dialysis access. At the age of 28, the patient was approved for another cadaveric kidney transplant. Following numerous blood transfusions (102 units of erythrocyte concentrates, 33 units of fresh frozen plasma, and 48 units of platelet concentrates) and two kidney transplantations, the patient became sensitized, with 3% current (7% highest) panel reactive antibodies. During the transplant procedure, the patient required hemodynamic stabilization by volume substitution, transfusion of filtered erythrocytes, and administration of high doses of norepinephrine. The postoperative course was complicated by methicillin-resistant *Staphylococcus aureus* bacteremia and accelerated acute cellular rejection. The patient responded well to the parenteral antibiotic and antirejection treatment and allograft function eventually stabilized.

Besides a higher fibrinogen level, preoperative coagulation parameters were always within the normal range. Radiologic imaging, performed due to the hemorrhagic shock and sepsis after the loss of the first kidney allograft, confirmed numerous TSC-associated lesions in the liver, lungs, and brain. Our patient was the only family member with clinical signs of TSC. Genetic analysis of other family members was not performed, because they had no signs of TSC and did not want to know their genetic background.

### Pathological findings

All tissue specimens were fixed and stained according to standard protocols with hematoxylin-eosin, and Van Gieson elastica, and immunohistochemically with primary antibodies including alpha-smooth muscle actin (SMA), Desmin, Human Melanoma Black (HMB)-45, Melan-A, Pan-Cytokeratin, D_2_40, estrogen receptor α, progesterone receptor, and cluster of differentiation (CD) 68.

**Figure 1:**
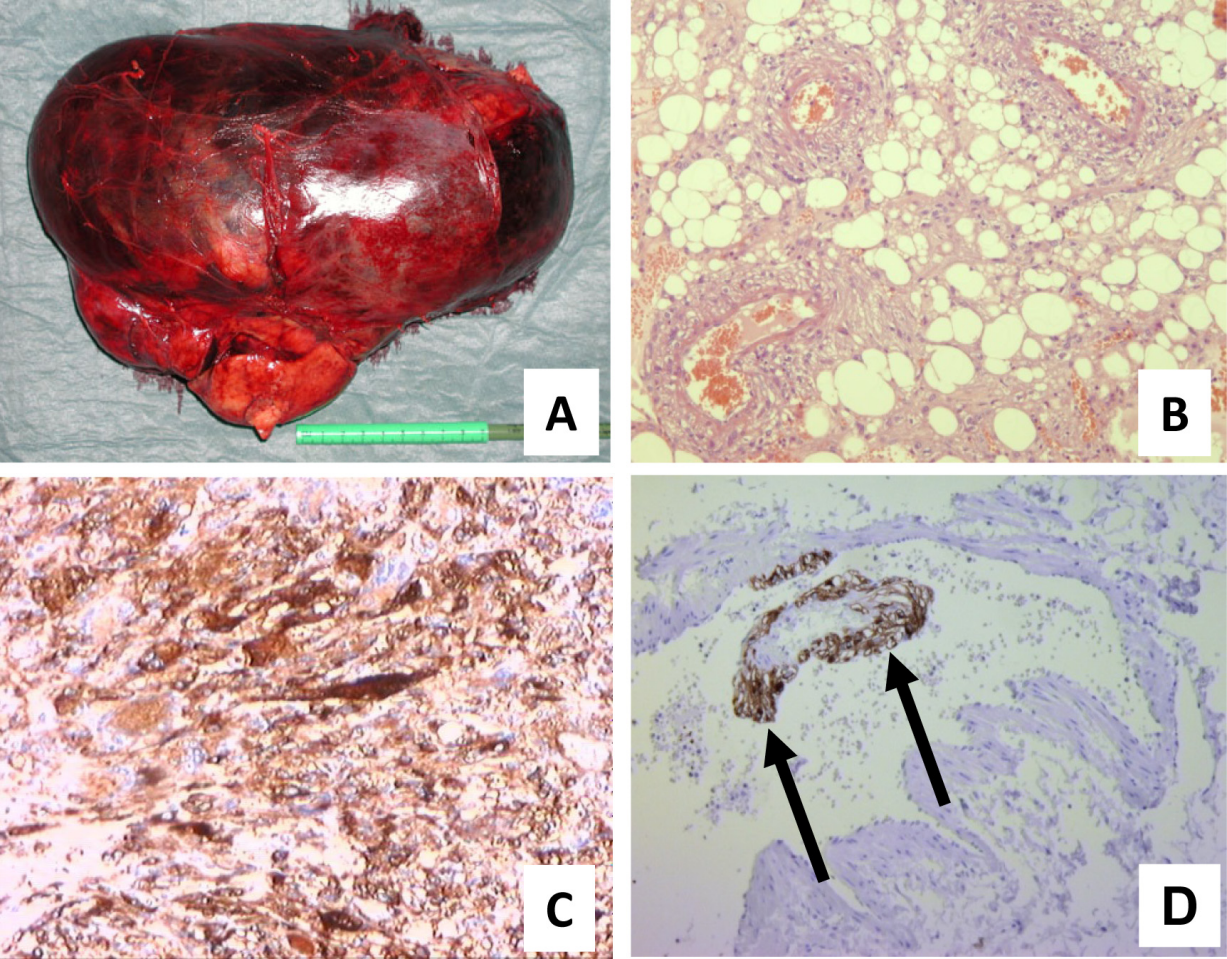
The angiomyolipomatous kidney and veins in the normal renal hilum with incompletely developed smooth muscle layers in the patient with severe form of tuberous sclerosis complex (TSC). **A.** The giant angiomyolipomatous right kidney (2.65 kg; 25 × 16 × 14 cm) compressed the renal pelvis and spread into the surrounding fatty tissue. **B.** The angiomyolipoma was composed of mature adipocytes, small and medium-sized blood vessels surrounded by collaretes of smooth muscle cells, with focally epithelioid appearance (hematoxylin-eosin, 100X). **C.** Spindle-shaped smooth muscle cells were positive for the neuroectodermal marker Human Melanoma Black (HMB)-45 (200X). **D.** The smooth muscle layer of veins in the normal renal hilum was incompletely developed and showed a focally positive expression of HMB-45 (100X).

*Kidney specimens.* Macroscopically, both kidneys were enormously enlarged, and the normal tissue was almost completely replaced by tumor tissue with areas of necrosis and hemorrhage, which compressed the renal pelvis and spread into the surrounding fatty tissue (Figure 1A). Histological and immunohistochemical findings corresponded to angiomyolipoma. Spindle cells were positive for SMA, HMB-45, and Melan-A (Figures 1B-D). The smooth muscle layer of veins in the normal renal hilum was incompletely developed (Figure 1D).

**Figure 2:**
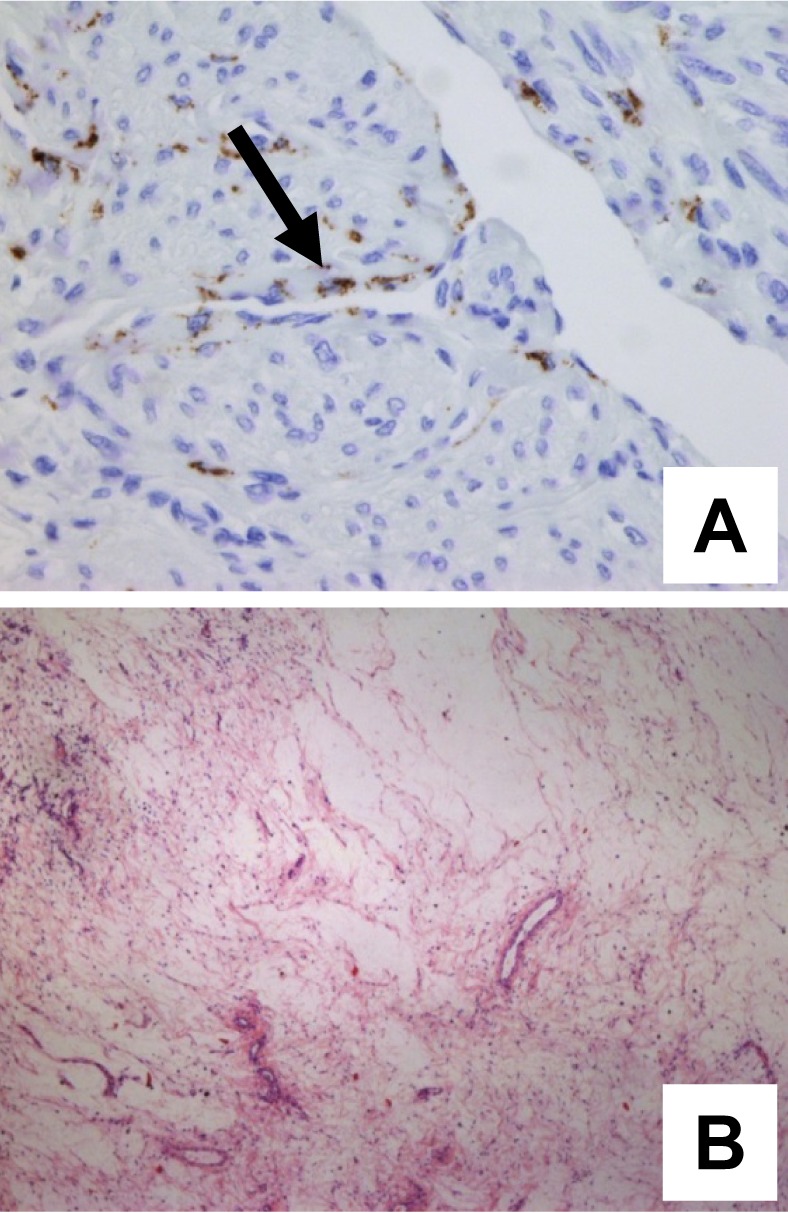
Tuberous sclerosis complex (TSC)-associated lesions found in the myometrium and ovaries of the patient. Immunohistochemistry revealed suspicious foci of uterine lymphangioleiomyomatosis, with Human Melanoma Black (HMB)-45 positivity in cells of vascular vessel walls. **A.** The arrow shows HMB-45 positive cells (400X). **B.** The left ovary was massively edematous and contained foci of abnormal lymphatic vessel proliferation (hematoxylin-eosin, 40X).

*Hysterectomy and salpingo-oophorectomy specimen*s. The uterus had a thickened endometrium showing simple hyperplasia. The myometrium had foci of lymphangioleiomyomatosis (LAM), positive for HMB-45 and D_2_40 staining (Figure 2A; data not shown). Both ovaries were enlarged and contained multiple cysts, most likely involuting follicular cysts. The dominant finding in the left ovary was a massive edema and foci showing abnormal lymphatic vessel proliferation (Figure 2B).

### Molecular genetic analysis

All 21 coding exons of the *TSC1* gene and all 41 coding exons of the *TSC2* gene, including the adjacent intronic sequences, were amplified using polymerase chain reaction (PCR) on genomic DNA extracted from peripheral leukocytes. The amplicons were directly sequenced by capillary electrophoresis using the Big Dye technology (Life Technologies, Darmstadt, Germany). For comparison and assessment of variants, the National Center for Biotechnology Information (NCBI) database (dbSNP, Bethesda MD, USA), the Exome Variant Server (NHLBI GO Exome Sequencing Project (ESP), Seattle, WA, USA), the Exome Aggregation Consortium (ExAC, Cambridge, MA, USA), the Human Gene Mutation Database (HGMD® professional release, Cardiff, UK), and the TSC locus-specific sequence variation database (LOVD, Leiden, The Netherlands) were used. cDNA reference sequences were NM_000368.4 for *TSC1* and NM_000548.3 for *TSC2*. According to historical convention, the exons of *TSC2* were numbered from the first coding exon. For the *in silico* prediction of variants with unknown clinical significance, algorithms of PolyPhen-2, Sorts Intolerant From Tolerant (SIFT), Align-Grantham Variation Grantham Deviation (Align-GVGD), MutationTaster, Single Nucleotide Polymorphism Database & Gene Ontology (SNPs&GO), MutPred, MutationAssessor, and Functional Analysis Through Hidden Markov Models (FATHMM) were applied. For the detection of genomic deletions and duplications, a quantitative analysis of all 23 *TSC1* exons and all 42 *TSC2* exons was carried out using Multiplex Ligation Probe Amplification (MLPA; SALSA-Kit P124-C1, 0112, and P046-C1, 1011, MRC-Holland, Amsterdam, The Netherlands).

A heterozygous *TSC2* c.3599G>C (p.R1200P) (exon 29) variant was detected in patient’s leukocyte DNA. No other variant or genomic deletion or duplication was identified in either *TSC1* or *TSC2*. DNA samples from the parents or other family members were not available for testing.

### Functional assessment of the TSC2 c.3599G>C (p.R1200P) variant

An expression construct for the *TSC2* c.3599G>C (p.R1200P) variant was derived by site-directed mutagenesis (SDM) of the wild-type *TSC2* expression construct (1). The construct was verified by sequencing the complete open reading frame; no additional nucleotide changes were introduced during the SDM procedure. Functional assessment was performed as described previously (1). The tuberin p.R1200P variant (R1200P) was compared with wild-type tuberin, the pathogenic p.R611Q (R611Q) variant, cells not expressing tuberin (TSC1/S6K) and cells transfected with the pcDNA3 expression vector only (mock). A p70 S6 kinase (S6K) reporter construct and *TSC1* expression construct were included in each transfection mixture, except for the mock. The total S6K signal measured by immunoblotting was used to estimate the relative transfection efficiency for each variant construct. The ratio of the signals for T389-phosphorylated S6K and total S6K (T389/S6K ratio) was used to estimate TORC1 activity. The TSC2, TSC1, S6K, and T389-phosphorylated S6K signals were estimated by immunoblotting in four independent transfection experiments.

**Figure 3:**
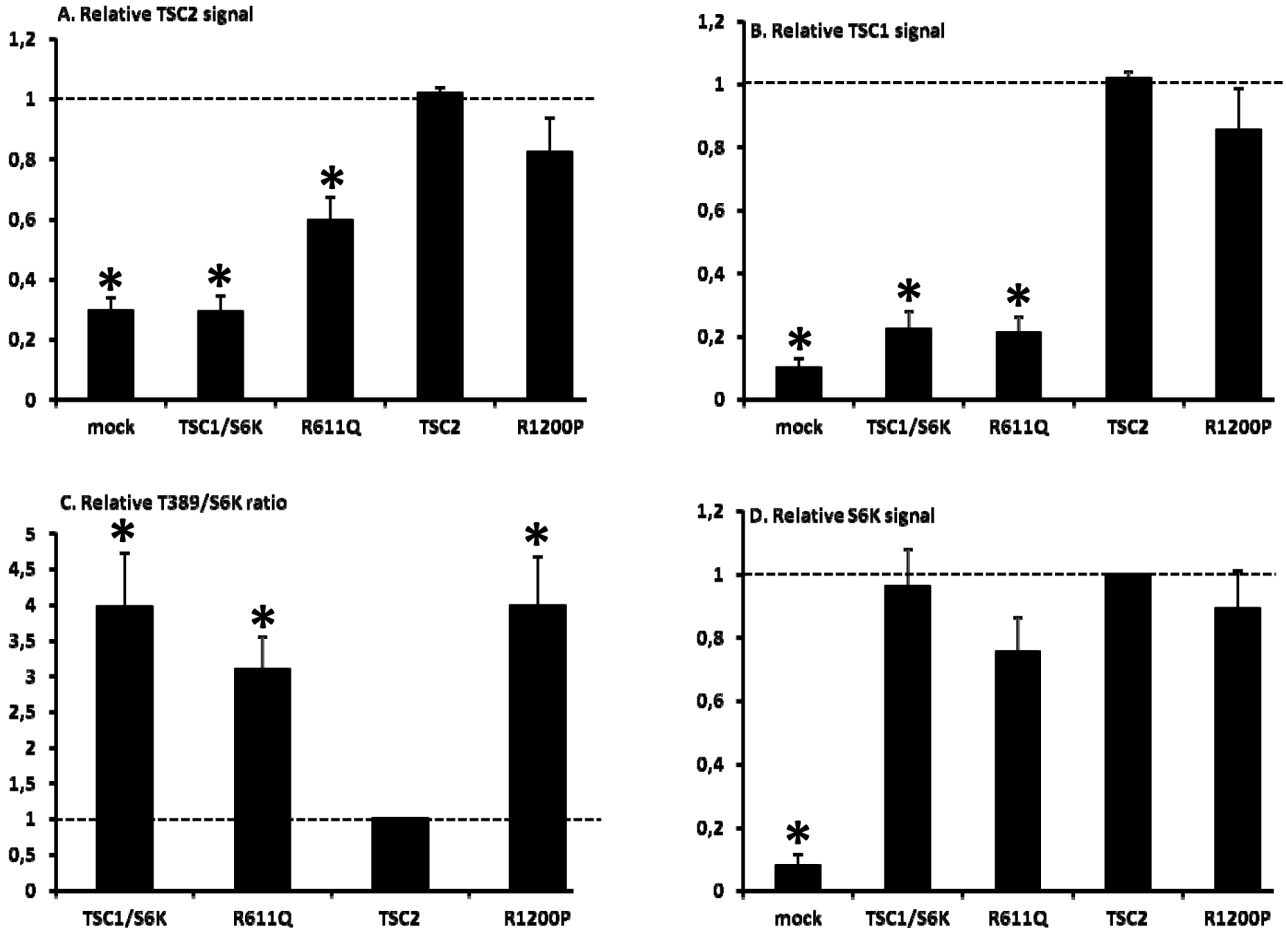
Functional assessment of the tuberous sclerosis complex (TSC) *TSC2* c.3599G>C (p.R1200P) variant showed a significantly increased T389-phosphorylated p70 S6 kinase/ total p70 S6 kinase (T389/S6K) ratio. The signals for TSC2, TSC1, total S6K (S6K), and T389-phosphorylated S6K (T389) were determined per variant, relative to the wild-type control (TSC2) in four independent transfection experiments. The mean TSC2 (**A**), TSC1 (**B**), and S6K (**D**) signals, and mean T389/S6K ratio (**C**) are shown for each variant. In each case the dotted line indicates the signal or ratio for wild-type TSC2 ( = 1.0). Error bars represent the standard error of the mean; variants that were significantly different from the wild-type control (TSC2) are indicated with an asterisk (*P* < 0.050; Student's *t* test). Amino acid changes are given according to *TSC2* cDNA reference transcript sequence NM_000548.3.

Functional assessment of the *TSC2* c.3599G>C (p.R1200P) variant indicated that the variant disrupted TSC complex function and was, therefore, likely to be pathogenic. The T389/S6K ratio was significantly increased, indicating impaired inhibition of TORC1 (Figure 3).

## DISCUSSION

We described the case of a female patient with serious TSC-associated complications, including mental retardation, recurrent hemorrhage, and the need for renal replacement therapy due to angiomyolipomas. At the time when angiomyolipomas were detected in our patient, the beneficial effects of mTOR inhibitors had not yet been established and kidney preserving procedures, such as arterial embolization or partial nephrectomy, were not feasible (2,3,7). Although angiomyolipomas are usually benign tumors, sometimes they can be aggressive, such as epithelioid angiomyolipomas (11). In our patient, pathohistological analysis revealed foci of epithelioid cells throughout the tumors. Giant angiomyolipomas completely replaced both kidneys and induced recurrent retroperitoneal bleeding.

Hemorrhage is known to be a major complication of these vascular tumors (3,5,7). Uterine lesions associated with TSC, such as lymphangioleiomyomatosis, angiomyoma or angiomyolipoma, may lead to bleeding disturbances (12). In our patient, metrorrhagia required hysterectomy, and postoperative hemorrhage caused the loss of two kidney allografts. We did not find any report on such bleeding complications after bilateral nephrectomy, even during long-term follow-up of TSC patients with a functional transplant. As there was no identifiable cause, we suspected that these postoperative bleeding episodes could be related to a multifocal anomaly in the development of the blood vessel wall.

The discovery of the molecular function of *TSC1* and *TSC2* and their involvement in TORC1 signaling provided tremendous insight into the pathogenesis of the disease. Hyperactive TORC1 signaling was shown to lead to abnormal vasculogenesis in TSC (4,13). The formation of arterial aneurysms in many organs is probably facilitated by abnormal elastin-poor structures in blood vessel walls in TSC-associated lesions (14). In our patient, the finding of focal expression of HMB-45 in vessel walls in the vicinity of the renal tumor indicated a multifocal growth pattern of angiomyolipoma. We noticed the characteristic co-expression of melanocytic (HMB-45, Melan A), histiocytic (CD68), and smooth muscle (SMA) markers (data not shown), which supports the unique nature of angiomyolipoma as a tumor with the ability for different phenotypic and immunotypic presentations (14). The abnormal ovarian lymphatic vessel proliferation, massive ovarian edema, multiple cystic formations in both ovaries, and uterine foci of proliferating LAM cells (abnormal smooth muscle-like cells) in our patient indicated the presence of generalized TSC-associated lesions. They could be the result of multicentric LAM cell proliferation, leading to the formation of fluid-filled cystic structures and hamartomas. LAM cells have heterogeneous HMB-45 reactivity, which has been related to the level of tuberin expression. It has been shown that an epigenetic alteration of *TSC2* can cause the loss of tuberin in TSC cells (15). *TSC2*-deficient smooth-muscle cells have a higher potential for migration than normal cells *in vitro* (15). A better understanding of the role of hamartin and tuberin in cell mobility and adhesion will further improve insight on the development of tumor deposits (4,14). Pharmacological therapy should target both the proliferation and migration of LAM cells in TSC. mTOR inhibitors, which have the ability to decrease the number of LAM cells in blood and urine, are recognized as therapeutic agents for the prevention of angiomyolipoma growth and kidney function loss (3,16).

According to the current diagnostic criteria, a diagnosis of TSC can be established clinically or genetically through the identification of a pathogenic *TSC1* or *TSC2* mutation (2). In families with several affected members, mutations are equally distributed between *TSC1* and *TSC2*. In our patient, molecular genetic examination of DNA in peripheral leukocytes identified the heterozygous variant c.3599G>C, (p.R1200P) in exon 29 of the *TSC2* gene. MLPA analysis did not reveal any genomic deletions or duplications in either *TSC1* or *TSC2*. The majority of TSC cases are sporadic (17). Sporadic cases mostly have a *TSC2* mutation, with *TSC2* mutations being associated with more severe forms of TSC disease (18). Our patient was the only family member with clinical signs of TSC. However, we can only speculate that her case was sporadic because other family members refused the testing.

The *TSC2* c.3599G>C (p.R1200P) variant has been included in the tuberous sclerosis database (LOVD), but without any information on the patients’ clinical manifestations. The variant is not included in the ESP or ExAC databases. The variant leads to the exchange of a highly conserved basic amino acid with a hydrophobic amino acid with moderate physicochemical differences. All the algorithms tested predicted a likely pathogenic impact on protein structure and function. Functional assessment demonstrated a significantly increased ratio of T389-phosphorylated S6K: total S6K (T389/S6K ratio) compared to wild-type TSC2, but not significantly different from the T389/S6K ratio of a known pathogenic variant, *TSC2* c.1832G>A (p.R611Q), indicating impaired inhibition of TORC1. Another variant in the same codon, c.3598C>T (p.R1200W), has been identified in several TSC patients, some of whom had a mild phenotype and reduced risk of infantile spasms (19). The observation that the p.R1200W variant also affects the tuberin-dependent inhibition of TORC1 (1) highlights the importance of amino acid position 1200 for tuberin function. Our study was limited by the fact that TSC with end-stage renal disease is a rare condition and we did not find any similar cases in the literature. Also, the genetic background was not further investigated because other family members refused genetic analysis.

This case report highlights the association of the pathogenic missense variant (p.R1200P) in the *TSC2* gene with a severe clinical course of TSC. The development of giant angiomyolipomas and generalized TSC-associated lesions caused organ loss and life-threatening hemorrhage. After many years of difficult treatment, our patient was submitted to third kidney transplantation with a successful outcome. Further research is needed to determine the extent of TSC-associated lesions and clinical manifestations related to this pathogenic missense variant.
